# Expression of different survivin variants in gastric carcinomas: first clues to a role of survivin-2B in tumour progression

**DOI:** 10.1038/sj.bjc.6600153

**Published:** 2002-03-04

**Authors:** A Krieg, C Mahotka, T Krieg, H Grabsch, W Müller, S Takeno, C V Suschek, M Heydthausen, H E Gabbert, C D Gerharz

**Affiliations:** Institute of Pathology, Heinrich Heine-University, Moorenstr 5, D-40225, Duesseldorf, Germany; Institute of Immunobiology, Heinrich Heine-University, Moorenstr 5, D-40225, Duesseldorf, Germany; Center for Applied Medical Informatics, Heinrich Heine-University, Moorenstr 5, D-40225, Duesseldorf, Germany

**Keywords:** alternative splicing, apoptosis, IAPs, RT–PCR, survivin

## Abstract

Survivin is a novel member of the inhibitor of apoptosis family and determines the susceptibility of tumour cells to pro-apoptotic stimuli. Recently, we identified two novel alternative splice variants of survivin, differing in their anti-apoptotic properties: whereas the anti-apoptotic potential of survivin-ΔEx3 is preserved, survivin-2B has lost its anti-apoptotic potential and may act as a naturally occurring antagonist of survivin. Because the *in vivo* expression of these alternative splice variants has not been explored so far, we analysed gastric carcinomas of different histological subtypes, grades and stages. Since no antibodies are currently available to determine the novel splice variants, quantitative reverse transcriptase polymerase chain reaction was performed, using RNA samples obtained from 30 different gastric carcinomas. Polymerase chain reactions products were quantified by densitometric evaluation. We found that all gastric carcinomas, irrespective of their histological types, grades or stages, express survivin-ΔEx3, survivin-2B and survivin, the latter being the dominant transcript. Comparing the disease stages I+II with III+IV, expression of survivin and survivin-ΔEx3 remained unchanged. In contrast, a significant (*P*=0.033) stage-dependent decrease in the expression of survivin-2B became evident. Our study demonstrates for the first time the expression of alternative splice variants in gastric carcinomas and provides a first clue to a role of survivin-2B in tumour progression.

*British Journal of Cancer* (2002) **86**, 737–743. DOI: 10.1038/sj/bjc/6600153 www.bjcancer.com

© 2002 Cancer Research UK

## 

Increasing resistance to programmed cell death by an imbalance between pro-apoptotic and anti-apoptotic proteins plays a critical role during tumorigenesis and tumour progression, facilitating the accumulation of transforming mutations and promoting evasion of tumour cells from immunosurveillance (Rudin and Thompson, 1997). A number of gene products with anti-apoptotic potential is known to modulate tumour cell viability and resistance to programmed cell death (Thompson, 1995; Chao and Korsmeyer, 1998; Fadeel *et al*, 2000). Recently, the inhibitor of apoptosis protein (IAP) family has been identified that is characterised by one or multiple domains designated as baculoviral IAP repeats (BIRs) (Crook *et al*, 1993; LaCasse *et al*, 1998). The BIR domain is considered to be essential for the anti-apoptotic potential of IAPs, which – inter alia – directly bind and inhibit terminal effector caspases (Roy *et al*, 1997; Deveraux and Reed, 1999).

Survivin was identified as a structurally unique member of the IAP family that contains only a single BIR domain (Ambrosini *et al*, 1997). Acting at the interface between apoptosis and mitosis, survivin was shown to be both a chromosomal passenger protein (Skoufias *et al*, 2000) and to prevent programmed cell death by inhibiting the actions of caspase-3 and caspase-7 (Tamm *et al*, 1998; Shin *et al*, 2001). Expression of survivin was found in proliferating foetal tissues, but not in most differentiated adult tissues (Ambrosini *et al*, 1997). Remarkably, increased survivin expression was observed in the most common human neoplasms, including oesophageal cancer, gastric cancer, colorectal carcinoma, non-small cell lung cancer, bladder cancer, neuroblastoma and lymphoma (Adida *et al*, 1998, 2000; Kawasaki *et al*, 1998; Lu *et al*, 1998; Monzo *et al*, 1999; Swana *et al*, 1999; Sarela *et al*, 2000; Kato *et al*, 2001). Expression of survivin may enhance tumour cell viability during tumour progression and enable the neoplastic cells to overcome the cytotoxic effects of anticancer drugs. As recently shown by our group (Mahotka *et al*, 1999), two novel alternatively spliced survivin variants, i.e. survivin-ΔEx3 and survivin-2B, may be relevant for the fine tuning of survivin actions: survivin-ΔEx3, which lacks exon 3, exhibits pronounced anti-apoptotic activity, whereas survivin-2B, which contains a part of intron 2 as an additional cryptic exon, has largely lost its anti-apoptotic activity. Survivin variants have also been detected in other species in the meantime (Conway *et al*, 2000; Wenzel *et al*, 2000), suggesting an evolutionarily conserved mechanism for the regulation of survivin actions by alternative splicing.

Whereas expression of survivin has previously been observed in gastric cancer (Lu *et al*, 1998), no data are available so far on the role of survivin-ΔEx3 and survivin-2B in this cancer type. Therefore, we quantified the expression levels of the different survivin variants in a panel of 30 gastric carcinomas. Because no antibodies are currently available that discriminate between the different survivin variants on the protein level, we used a survivin-specific reverse transcriptase polymerase chain reaction (RT–PCR) for our analysis. This study provided first *ex vivo* evidence for a role of survivin-2B in the progression of gastric cancer.

## MATERIALS AND METHODS

### Patients and specimens

Tissue samples of 30 consecutive gastric carcinomas were obtained from patients who had undergone gastrectomy. The tumour specimens were immediately flash frozen in liquid nitrogen and stored at −80°C until total RNA extraction or protein isolation was performed. Tumour typing, grading and staging were performed according to the principles outlined by the WHO (Watanabe *et al*, 1998) and the UICC (Sobin and Wittekind, 1997). Additional clinicopathological parameters such as age, sex, lymphatic vessel invasion, blood vessel invasion, *Helicobacter pylori* colonisation and density of the chronic inflammatory cell infiltration in the tumour-adjacent normal gastric mucosa (according to the Sydney System (Dixon *et al*, 1996)) were defined for all patients.

### RNA–extraction

Total RNA of the tumours and paired non-neoplastic tissue samples was isolated by acid guanidium thiocyanate phenol chloroform extraction with minor modifications as previously described (Chomczynski and Sacchi, 1987).

### Reverse transcription and polymerase chain reaction

Total RNA (2 μg) was reversely transcribed in a final 30 μl reaction volume using 5 U AMV reverse transcriptase (Promega, Heidelberg, Germany), 1×RT buffer (Promega), 20 U of RNase inhibitor RNAsin (Promega), 25 μM of each deoxynucleotide triphosphate (Qiagen, Hilden, Germany) and 10 pmol of each sequence-specific RT primer. Because survivin mRNA has to be distinguished from effector cell protease receptor-1 (EPR-1) mRNA, a naturally occurring antisense-mRNA of survivin which might bias survivin amplification (Zaman and Conway, 2000), we used the primer 5′-AGG AAC CTG CAG CTC AGA-3′, corresponding to nucleotides 914–931 of the survivin antisense strand (accession no NM_001168). For GAPDH-specific cDNA synthesis, the antisense primer 5′-CTC CTG GAA GAT GGT GAT GG-3′, corresponding to nucleotides 251–270 of the GAPDH antisense strand (accession no J04038) was used. Reverse transcription of survivin and GAPDH was performed in the same reaction to avoid variations in efficiency of cDNA synthesis. The specific RT reaction mixtures were incubated at 50°C for 1 h. PCR amplification was performed in a final volume of 50 μl containing 3 μl first strand cDNA solution, 2.5 U of Taq polymerase, 1×PCR-buffer, 10 μl Q-solution (except GAPDH amplification), 25 μM of each dNTP (all Qiagen) and 25 pmol of each 3′ and 5′ sequence specific oligonucleotide primer.

To determine the saturation phase of RT–PCR amplification, we started with a cycle titration for survivin and GAPDH. Reactions were stopped after 15, 20, 25, 30, 35 and 40 cycles, and the PCR products of the different cycle numbers were electrophoresed on 3% agarose gels containing ethidium bromide. The signal intensity of amplification products was quantified by densitometry as described below. PCR was then performed for all cDNA samples with 35 (survivin) or 25 cycles (GAPDH), which did not reach the saturation phase and permitted the densitometric quantification and comparison of amplification product levels.

Survivin-PCR amplification was performed on a PTC-100 Thermal controller (Biozym Diagnostic, Hess, Oldendorf, Germany) with initial denaturation at 95°C for 2 min, followed by 35 cycles of denaturation at 94°C for 30 s, annealing for 1 min at 58°C, extension at 72°C for 1 min and a final extension at 72°C for 5 min. GAPDH–PCR amplification conditions were identical to those described above except annealing at 64°C and 25 cycles. Primers were designed to amplify the entire coding region of survivin: 5′-GCA TGG GTG CCC CGA CGT TG-3′ (forward; corresponding to position 48–67 of the survivin mRNA; accession no NM_001168) and 5′-GCT CCG GCC AGA GGC CTC AA-3′ (reverse). RT–PCR for GAPDH as a constitutively expressed gene was performed using the primers 5′-ACG GAT TTG GTC GTA TTG GGC G-3′ (forward; corresponding to position 59–80; accession no J04038) and 5′-CTC CTG GAA GAT GGT GAT GG-3′ (reverse). PCR products were electrophoresed on 3% agarose gels containing ethidium bromide and visualised under UV-transillumination.

All RT–PCR amplifications were carried out in duplicate to confirm reproducibility. To ensure that amplification of survivin and GAPDH was specific, PCR bands were excised from agarose gels and isolated using the QIAquick gel extraction kit (Qiagen). The purified PCR-products were sequenced using the ABI-Prism BigDye Terminator Cycle Sequencing Kit (Applied Biosystems, Weiterstadt, Germany) and the oligonucleotides designed for PCR amplification. Sequence analysis was performed with a ABI-Prism 310 sequencer (Applied Biosystems).

### Quantification of PCR–products and statistical analysis

Electrophoresed and ethidium bromide stained PCR products from two independent RT–PCR reactions were recorded under UV-transillumination by the Gel-Doc 1000 apparatus (Bio-Rad, München, Germany) and quantified by densitometric evaluation of signal intensity using the Kodak Digital Science 1D software. GAPDH mRNA levels were used to normalise the mRNA levels of the different survivin variants, calculating relative mRNA levels as the ratios between survivin variants and GAPDH.

### Immunoblotting

Protein extracts from four arbitrarily selected gastric carcinomas were isolated by disrupting flash frozen tissue samples in lysis buffer (100 mM NaCl, 10 mM Tris-HCl pH 7.6, 1 mM EDTA pH 8, 1% NP40 and protease-inhibitor). Protein aliquots (100 μg) were electrophoresed through 15% SDS-polyacrylamide gels at 70 mA for 4 h. Blotting to Optitran BA-S85 nitrocellulose membranes (Schleicher & Schuell, Dassel, Germany) was performed for 1.5 h at 650 mA in a tank of transfer buffer pH 8.3 (25 mM Tris-HCl, 192 mM Glycin, 20% Methanol) using the Hoefer TE series Transphor electrophoresis Unit (Hoefer Scientific instruments, San Francisco, CA, USA). To verify transfer efficiency and protein integrity, nitrocellulose membranes were stained with Ponceau S 0.2%. The membranes were blocked over night in blocking buffer (100 mM Tis-HCl, pH 7.5, 150 mM NaCl, 0,2% Tween 20) plus 3% non-fat dry milk and 1% BSA, incubated for 2 h at room temperature with the polyclonal rabbit anti-human survivin antibody SURV 11-A (Alpha Diagnostic International Inc, San Antonio, TX, USA; dilution: 1 : 1000), washed, and incubated with a 1 : 2000 dilution of horseradish peroxidase-linked donkey anti-rabbit antibody for 1 h at room temperature. After washing, protein detection was performed by incubation with Lumi-Light substrate (Roche, Mannheim, Germany). Equal amounts of the loaded samples were confirmed by β-actin detection with the monoclonal mouse anti-human β-actin antibody (Sigma-ldrich, Deisenhofen, Germany; clone AC-15). Data on X-ray films were quantified by densitometry.

### Statistical analysis

For statistical analysis, disease stages were defined according to the principles outlined by the UICC (Sobin and Wittekind, 1997) and disease stages I and II were compared with disease stages III and IV. Statistical analysis was performed using the SPSS 9.0 software package (SPSS Inc., Chicago, IL, USA). The expression of the different survivin-variants in cancer tissues was compared by the Wilcoxon-test. Correlation between the expression levels of survivin variants and clinicopathological variables was examined using the nonparametric Mann–Whitney-test. A two-tailed *P*-value less than 0.05 was considered to indicate statistical significance.

## RESULTS

### Expression of survivin variants

**Figure 1:**
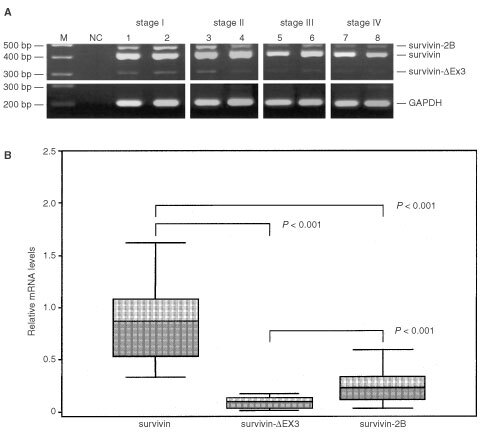
RT–PCR amplification (**A**) and relative (GAPDH-normalised) mRNA levels (**B**) of survivin and its alternative splice variants in gastric cancer. (**A**) Two μg of total RNA was reversely transcribed and amplified by polymerase chain reaction. PCR-products were electrophoresed and visualised by ethidium bromide staining. Four arbitrarily selected examples of each UICC disease stage are shown. M, molecular weight marker; NC, negative control (H_2_O); survivin (431 bp); survivin-2B (500 bp); survivin-ΔEx3 (329 bp). (**B**) Survivin is the dominant transcript. *P*-values were calculated by the Wilcoxon-test.

Expression of survivin and its alternative splice variants (survivin-ΔEx3 and survivin-2B) was detectable in all gastric cancer specimens (*n*=30), irrespective of their histological types, grades and stages (Figure 1A

). Because we had applied cycle numbers for PCR amplification of survivin and GAPDH that did not reach the saturation phase, a densitometric evaluation and comparison of the mRNA levels between different tissue samples was feasible. As shown in Figure 1B, survivin was the predominant transcript variant, whereas only low levels of survivin-ΔEx 3 and survivin-2B were observed. The differences of expression between the survivin variants were statistically significant (*P*<0.001).

**Figure 2:**
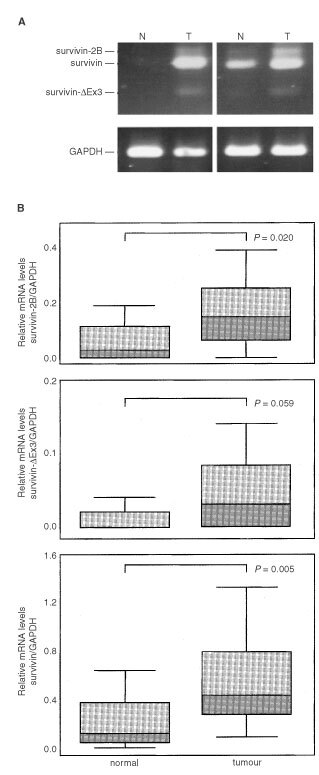
RT–PCR-amplification (**A**) and relative (GAPDH-normalised) mRNA levels (**B**) of survivin variants in paired samples of non-neoplastic (N) and neoplastic (T) gastric tissue. (**A**) In eight out of 14 non-neoplastic tissue samples, no or barely detectable levels of survivin mRNA levels were observed. In each cancer tissue sample, survivin expression level exceeded that of the corresponding non-neoplastic tissue sample. GAPDH served as internal control. (**B**) Expression of survivin and survivin-2B – but not survivin-ΔEx3 - are elevated in gastric carcinomas. Two-tailed *P*-values were calculated by the Mann–Whitney-test.

Fourteen matched pairs of non-neoplastic and neoplastic gastric tissue samples were compared for their expression of survivin variants. In eight out of 14 non-neoplastic tissue samples, no or barely detectable levels of survivin mRNA levels were observed. Survivin expression in each cancer tissue sample exceeded that of the matched non-neoplastic tissue sample (Figure 2A

), the overall expression levels in cancer being significantly higher (*P*=0.005) when compared to non-neoplastic tissues (Figure 2B). Whereas survivin-ΔEx3 expression did not significantly differ between non-neoplastic and neoplastic tissue samples (*P*=0.059), survivin-2B expression was significantly (*P*=0.02) elevated in gastric carcinomas (Figure 2B).

### Stage-dependent decrease in survivin-2B expression

**Figure 3:**
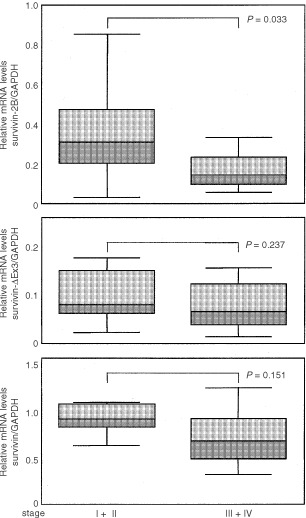
Relative (GAPDH-normalised) mRNA levels of the different survivin variants in gastric carcinomas of different stages. A significant decrease in the expression of survivin-2B is found in advanced disease stages. Two-tailed *P*-values were calculated by the Mann–Whitney-test.

**Figure 4:**
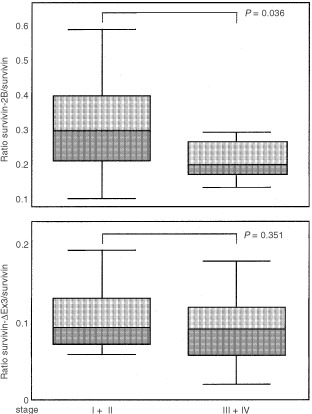
Ratio of relative mRNA levels between the different survivin variants in gastric carcinomas. Significant decrease of the survivin-2B/survivin ratio in advanced (III+IV) tumour stages. Two-tailed *P*-values were calculated by the Mann–Whitney-test.

Survivin expression has previously been reported to be related to prognosis of cancer patients (Adida *et al*, 1998, 2000; Kawasaki *et al*, 1998; Monzo *et al*, 1999; Swana *et al*, 1999; Sarela *et al*, 2000; Kato *et al*, 2001). Therefore, we analysed the stage-dependent expression of the different survivin variants, comparing low stages (I+II according to UICC (Sobin and Wittekind, 1997)) and advanced stages (III and IV according to UICC (Sobin and Wittekind, 1997)) of gastric cancer. As shown in Figure 3

, the expression levels of the anti-apoptotic survivin variants (i.e. survivin and survivin-ΔEx3) did not significantly change between low (I+II) and advanced (III+IV) tumour stages. In contrast, a significant (*P*=0.033) decrease in the expression of the non-anti-apoptotic survivin-2B variant became evident in advanced stages of gastric cancer. To ensure that the stage-dependent decrease of survivin-2B expression was not the result of a stage-dependent increase of GAPDH transcripts used as an external standard, we additionally calculated the ratios of mRNA levels between survivin-ΔEx3 or survivin-2B on one hand and survivin as an internal standard on the other hand. Because all survivin variants are derived from a common hnRNA precusor pool, these ratios are independent from a possible bias imposed by possible variations of GAPDH expression levels. The difference between these ratios (*P*=0.037) further confirmed the significant stage-dependent decrease of non-anti-apoptotic survivin-2B (Figure 4

).

### Correlation between the expression levels of survivin variants and clinicopathological parameters

To detect a possible correlation between the expression levels of survivin variants and other clinicopathological parameters, the Mann–Whitney-test for nonparametric data was performed. With the exception of the stage-dependent decrease in the expression of survivin-2B (see above), none of the investigated clinicopathological variables showed a statistically significant correlation with the expression levels of survivin and its alternative splice variants (Table 1

**Table 1:**
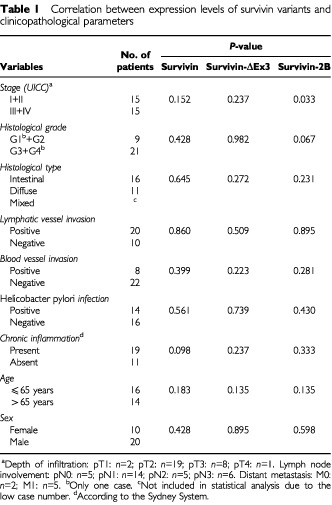
Correlation between expression levels of survivin variants and clinicopathological parameters

).

### Expression of survivin protein in gastric carcinomas

**Figure 5:**
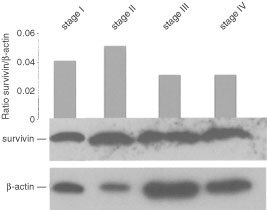
Expression of survivin protein in gastric carcinomas. One hundred μg protein of each tumour sample were assessed for survivin and β-actin expression. Grey bars reflect the relative expression levels of survivin as measured by densitometry.

Because no antibodies are available so far which recognise survivin-2B and survivin-ΔEx3, we could only analyse the expression of survivin protein in four arbitrarily selected gastric carcinoma samples that represent the UICC stages I to IV. All tissue samples showed survivin protein, but no uniform increase of survivin expression became evident in more advanced tumour stages (Figure 5

).

## DISCUSSION

In this study, we show that all tissue specimens of gastric cancer express the apoptosis inhibitor survivin and extend a previous report by Lu *et al* (1998) on survivin *protein* expression in 34.5% of gastric cancer cases to all cases of our study on the mRNA level, using the more sensitive RT–PCR technique. More importantly however, we demonstrate for the first time that the novel splice variants survivin-Ex3 and survivin-2B are expressed in *ex vivo* tumour samples of gastric cancer.

Recently, survivin has been identified as a structurally and functionally unique member of the inhibitor of apoptosis protein (IAP) family that is highly conserved during evolution (Wenzel *et al*, 2000) and involved in the regulation of both apoptosis and cell cycle progression (Li *et al*, 1998, 1999). At variance with the ubiquitous distribution of other IAPs in foetal and adult tissues, intensive survivin expression in proliferating foetal tissues contrasts with down-regulation in most differentiated adult tissues (Ambrosini *et al*, 1997). Most strikingly, however, expression of survivin was found in the most common human cancer types, which might promote both tumour progression and resistance to chemotherapy and irradiation. The molecular mechanisms by which survivin inhibits apoptosis are still under investigation. *In vitro* experiments have shown that survivin binds and inhibits the cell death effector proteases caspase-3 and caspase-7 (Tamm *et al*, 1998; Shin *et al*, 2001). Moreover, survivin was found to associate specifically with centromeres, microtubules of the mitotic spindle and Cdk4, thereby supposedly regulating the progression of foetal and neoplastic cells through mitosis (Li *et al*, 1998; Suzuki *et al*, 2000; Uren *et al*, 2000; Wheatley *et al*, 2001).

The complexity of survivin actions is further augmented by the expression of two functionally divergent alternative splice variants, i.e. survivin-ΔEx3 and survivin-2B. As recently demonstrated by our group (Mahotka *et al*, 1999), both splice variants exhibit structural alterations of their single BIR domain. Despite these structural modifications, the anti-apoptotic potential of survivin-ΔEx3 seems to be largely preserved, whereas survivin-2B exhibited a loss of anti-apoptotic properties and, therefore, might act as a naturally occurring antagonist of survivin (Mahotka *et al*, 1999). Although further experimental work is necessary to elucidate the functional properties of the different splice variants in more detail, first clues to the involvement of survivin-ΔEx3 and survivin-2B in the progression of gastric carcinoma can be derived from our present study.

In this study, we found mRNA expression of the anti-apoptotic variants survivin and survivin-ΔEx3 in all major histological types of gastric carcinomas, irrespective of their grading and staging. Analysis of matched pairs of non-neoplastic and neoplastic tissue samples revealed expression of both survivin variants in non-neoplastic gastric tissues and a significant increase in survivin – but not survivin-ΔEx3 – mRNA expression levels in cancer tissue. Especially the use of more sensitive RT–PCR has demonstrated survivin expression in other non-neoplastic tissues as well, including normal lung samples, endothelial cells and lymphocytes (Monzo *et al*, 1999; Tran *et al*, 1999; Kornacker *et al*, 2001). The low levels of survivin mRNA in non-neoplastic tissues argue against the assumption that increased survivin levels in gastric cancer might only be due to an increased proportion of lymphocytes. Our observations, therefore, further confirmed the ubiquitous increase of survivin expression in human cancer as previously reported for other tumour types as well (Adida *et al*, 1998, 2000; Kawasaki *et al*, 1998; Lu *et al*, 1998; Monzo *et al*, 1999; Swana *et al*, 1999; Sarela *et al*, 2000; Kato *et al*, 2001).

Increased survivin mRNA or protein expression has previously been reported to be a prognostic indicator of tumour progression in different types of human cancer (Adida *et al*, 1998, 2000; Kawasaki *et al*, 1998; Monzo *et al*, 1999; Swana *et al*, 1999; Sarela *et al*, 2000; Kato *et al*, 2001). The densitometric quantification of mRNA levels performed in our study, however, did not reveal a correlation between expression levels of survivin and the histological types, grades or stages of gastric carcinomas. This observation was further supported by Western blot analysis of arbitrarily selected gastric carcinomas, and is in accordance with a previous immunohistochemical analysis of gastric cancer (Lu *et al*, 1998), which failed to reveal a significant correlation between survivin expression and tumour depth, lymph node metastasis or disease stage. Increased expression of anti-apoptotic survivin, therefore, might be an early event in gastric carcinogenesis, as has formerly been suggested for survivin in other tumour types as well (Grossman *et al*, 1999).

In accordance with our observations in renal carcinoma cell lines (Mahotka *et al*, 1999), the mRNA levels of survivin-2B in gastric carcinomas were significantly lower than those of survivin. Moreover, the expression of survivin-2B significantly decreased in advanced stages of gastric cancer. These low levels of survivin-2B expression, however, might not reflect the actual significance of survivin-2B for the progression of gastric cancer. Thus, the stage-dependent downregulation of survivin-2B might affect the susceptibility to apoptosis induction in two ways. The decrease of survivin-2B transcripts could permit the generation of more anti-apoptotic survivin variants (i.e. survivin and survivin-ΔEx3), because all survivin variants originate from a common pool of a hnRNA percusor. Parallel to the decrease of survivin-2B, the anti-apoptotic effects of survivin could become even more predominant, if survivin-2B actually acts as a natural antagonist as suggested by Islam *et al* (2000).

In conclusion, our study presents first data on the *in vivo* expression of novel survivin splice variants in gastric carcinomas. Expression of survivin-2B – but not of survivin and survivin-ΔEx3 – was shown to be related to tumour progression in gastric cancer. These findings indicate for the first time that alternative splice variants of survivin may be involved in the fine tuning of survivin actions in gastric carcinomas.
